# Bryostatin-1 Attenuates Ischemia-Elicited Neutrophil Transmigration and Ameliorates Graft Injury after Kidney Transplantation

**DOI:** 10.3390/cells11060948

**Published:** 2022-03-10

**Authors:** Felix Becker, Linus Kebschull, Constantin Rieger, Annika Mohr, Barbara Heitplatz, Veerle Van Marck, Uwe Hansen, Junaid Ansari, Stefan Reuter, Benjamin Strücker, Andreas Pascher, Jens G. Brockmann, Trevor Castor, J. Steve Alexander, Felicity N. E. Gavins

**Affiliations:** 1 Department of General, Visceral and Transplant Surgery, University Hospital Münster, 48149 Münster, Germany; felix.becker@ukmuenster.de (F.B.); kebschull@googlemail.com (L.K.); constantin.rieger@uni-muenster.de (C.R.); Annika.Mohr@ukmuenster.de (A.M.); benjamin.struecker@ukmuenster.de (B.S.); andreas.pascher@ukmuenster.de (A.P.); Jens.Brockmann@ukmuenster.de (J.G.B.); 2 Gerhard Domagk Institute of Pathology, University Hospital Münster, 48149 Münster, Germany; barbara.heitplatz@ukmuenster.de (B.H.); Veerle.VanMarck@ukmuenster.de (V.V.M.); 3 Department of Molecular Medicine, Institute for Musculoskeletal Medicine, University Hospital Münster, 48149 Münster, Germany; Uwe.Hansen@ukmuenster.de; 4 Department of Neurology, Louisiana State University Health Sciences Center, Shreveport, LA 71130, USA; junaid.ansari@lsuhs.edu; 5 Division of General Internal Medicine, Nephrology and Rheumatology, Department of Medicine D, University Hospital of Münster, 48149 Münster, Germany; Stefan.Reuter@ukmuenster.de; 6 Aphios Corporation—R&D, Woburn, MA 01801, USA; tcastor@aphios.com; 7 Department of Molecular and Cellular Physiology, Louisiana State University Health Sciences Center, Shreveport, LA 71130, USA; 8 Department of Life Sciences, Centre for Inflammation Research and Translational Medicine (CIRTM), Brunel University London, Uxbridge UB8 3PH, UK

**Keywords:** kidney transplant, ischemia reperfusion injury, Bryostatin-1, translational research

## Abstract

Ischemia reperfusion injury (IRI) is a form of sterile inflammation whose severity determines short- and long-term graft fates in kidney transplantation. Neutrophils are now recognized as a key cell type mediating early graft injury, which activates further innate immune responses and intensifies acquired immunity and alloimmunity. Since the macrolide Bryostatin-1 has been shown to block neutrophil transmigration, we aimed to determine whether these findings could be translated to the field of kidney transplantation. To study the effects of Bryostatin-1 on ischemia-elicited neutrophil transmigration, an in vitro model of hypoxia and normoxia was equipped with human endothelial cells and neutrophils. To translate these findings, a porcine renal autotransplantation model with eight hours of reperfusion was used to study neutrophil infiltration in vivo. Graft-specific treatment using Bryostatin-1 (100 nM) was applied during static cold storage. Bryostatin-1 dose-dependently blocked neutrophil activation and transmigration over ischemically challenged endothelial cell monolayers. When applied to porcine renal autografts, Bryostatin-1 reduced neutrophil graft infiltration, attenuated histological and ultrastructural damage, and improved renal function. Our novel findings demonstrate that Bryostatin-1 is a promising pharmacological candidate for graft-specific treatment in kidney transplantation, as it provides protection by blocking neutrophil infiltration and attenuating functional graft injury.

## 1. Introduction

Organ shortage remains the cardinal problem in transplant medicine, and the use of organs with extended ischemia time is becoming routine, reflecting decreasing numbers of donated organs in contrast to increasing numbers of patients on the waiting list. In the US alone, there were (as of 2019) 101,353 patients on the kidney transplant waitlist, with 24,273 kidney transplants being performed annually [1].

Exposure to ischemia and reperfusion is an unavoidable event during organ transplantation and is directly linked to graft damage [2]. In the kidney, ischemia reperfusion injury (IRI) produces an inflammatory state that leads to acute kidney injury (AKI) and a high susceptibility to subsequent delayed graft function (DGF), a form of acute post-transplant graft dysfunction, which correlates with IRI severity [3,4]. Beyond short-term complications, the initial inflammatory response and DGF are critically linked to allograft immunogenicity and long-term graft function; thus, the severity of acute graft dysfunction predicts overall graft survival [5,6].

Renal tubular epithelial cells and especially vascular endothelial cells (ECs) are highly vulnerable to IRI [7,8]. Oxygen deprivation during cold storage results in inhibition of oxidative metabolism, generation of reactive oxygen species (ROS), and accumulation of metabolic waste products. Paradoxically, the restoration of blood flow during reperfusion further intensifies graft injury and induces cellular injury pathways ranging from cell activation to necrosis [9]. While the exact molecular responses in IRI remain complex and not fully understood, it is widely accepted that this array of pathways results in an innate inflammatory response in which neutrophils are the first leukocyte population to infiltrate the kidney, as early as 30 min after reperfusion [9,10]. Thus, neutrophils represent the primary mediators of early host-induced tissue injury and act as the ‘gatekeeper’ cell, reflecting their ability to orchestrate the influx of subsequent waves of leukocytes into the graft. This is an important concept in transplantation because limitation of initial neutrophil infiltration may reduce subsequent recruitment of additional inflammatory cells and more extensive, later phases of tissue damage [11]. In keeping with this concept, experimental strategies depleting neutrophils or inhibiting their early infiltration have been effective in reducing IRI and improving graft function and survival [10,11,12,13,14,15]. However, these strategies are largely confined to the treatment of the organ recipient following graft implantation.

Neutrophil recruitment into sites of injury or inflammation involves a multistep cascade and crosstalk between immune cells and ECs consisting of selectin-mediated rolling, integrin-mediated firm adhesion, and ultimately neutrophil trans-endothelial migration (TEM) [16]. In the sequence of TEM, EC second messengers, such as protein kinase C (PKC), and their pharmacological activation have gained recent attention since they may control several mechanisms governing neutrophil TEM, including cytoskeleton remodeling and integrity of EC junctional proteins [17].

Bryostatin-1 is a macrolide lactone isolated from the bryozoan *Bugula neritina*. It has been demonstrated that Bryostatin-1 activates the EC messenger PKC delta (δ) at low nano- to picomolar levels, thereby blocking neutrophil TEM in vitro [18]. Of significance, Bryostatin-1 can block these steps by activating PKCδ without downregulating PKCδ after sustained activation, which could lead to adverse effects, such as disruption of the EC barrier [18]. In fact, Bryostatin-1 has even been shown to enhance barrier integrity and to block cytokine-induced barrier alterations [19]. Therefore, Bryostatin-1 represents a promising pharmacological agent that may be able to ameliorate two hallmark events of renal IRI: neutrophil TEM and the breakdown of EC integrity.

Given the promising pharmacological profile of Bryostatin-1, we wanted to test its potential therapeutic value in the field of transplant medicine. Specifically, we have combined in vitro approaches with a relevant and translational in vivo porcine model to test the hypothesis that Bryostatin-1 provides protection against renal IRI by altering neutrophil–EC interactions.

## 2. Materials and Methods

### 2.1. Human Samples

After signed consent, blood was taken from control volunteers (male and female adults older > 18 years of age) and patients with sickle cell disease (SCD). Since SCD is known for its heightened endogenous neutrophil activation, neutrophils from SCD patients were used to recapitulate the IRI-elicited neutrophil activation in renal transplant recipients. The study was approved by the institutional review board of the Louisiana State University Health Science Center—Shreveport (LSUHSC-S, STUDY00000572 and STUDY00000261) and conducted in accordance with the Declaration of Helsinki. SCD patients were recruited upon routine clinical visits at the Feist-Weiller Cancer Center at LSUHSC-S, and volunteers who were pregnant were excluded from the study. All SCD patients were on chronic hydroxyurea therapy, and blood was obtained just before exchange transfusion. Hydroxyurea was started at 15 mg per kilogram of body weight per day and then escalated by 5 mg per kilogram every 12 weeks until the maximum tolerated dose was achieved based on peripheral blood counts. Patients were on partial exchange transfusions every two weeks. Patients with acute infection or other chronic blood-borne diseases (HIV, Hepatitis B/C) were excluded from the study. Studies were performed blinded and randomized.

### 2.2. Experimental Design for In Vitro Studies

Transwell systems, with confluent human umbilical vein endothelial cell (HUVEC) monolayers, were used to study endothelial barrier function and cellular transmigration under normoxic or hypoxic conditions. Briefly, HUVECs were treated with varying concentrations of Bryostatin-1 in a modified PET (Polyethylene glycol, 100% Ethanol, Tween 80) formulation (1, 10, 100 nM, Aphios Corporation, Woburn, MA, USA) or vehicle (PET) and incubated for 24 h either at 4 °C or 37 °C in normoxic or hypoxic conditions (see below). After this time, HUVECs were aspirated to remove Bryostatin-1. Neutrophils or fluorescein isothiocyanate (FITC)-dextran were then added to the inserts (see below), and the following assays were performed: neutrophil TEM, FITC-dextran permeability, or neutrophil TEM + FITC-dextran permeability (see below). In some experiments, neutrophils were subjected to ex vivo activation with tumor necrosis factor alpha (TNF-α, 10 ng/mL, 30 min).

### 2.3. Maintenance of HUVECs

HUVECs were isolated from normal term pregnancies as previously described [20]. HUVECs were incubated in vascular endothelial growth factor (VEGF) endothelial cell culture medium with added growth factors and supplements as provided by the company (VascuLife^®^ VEGF Endothelial Medium Complete Kit, Lifeline, Frederick, MD, USA). HUVEC media was further supplemented with 10% fetal calf serum (FCS) and antibiotics (penicillin/streptomycin/amphotericin B). HUVECs were cultured in 1% gelatin coated flasks and incubated at 37 °C with 5% CO_2_. Before experiments were conducted, HUVECs were transferred to transwell inserts coated with fibronectin (10 µg/mL) at 32,000 cells/insert and grown for 72 h to reach confluency. For all following experiments, HUVECs transwell inserts were used with 24-well plates (Corning, Corning, NY, USA).

### 2.4. Isolation of Human Neutrophils

Human neutrophils were isolated from healthy individuals or SCD patients and prepared as described previously [21]. Initially, acid citrate dextrose containing blood was centrifuged at 800 rpm for 20 min at room temperature after collection, as described previously [21]. The plasma was removed, and 10 mL 1 X PBS was slowly layered on top of the remaining blood followed by 8 mL 6% dextran (Spectrum Chemical, New Brunswick, NJ, USA) without disturbing the bottom layer in a 50 mL test tube. Thereafter, the test tube was slowly mixed and allowed to sediment for 20 min. The pink leukocyte layer at the top was collected from the test tube and carefully layered over 10 mL Histopaque 1077 (Sigma-Aldrich, St. Louis, MO, USA). This was followed by centrifugation at 1500 rpm for 30 min at room temperature. The formed supernatant was aspirated, and the remaining pellet was resuspended in 9 mL ice-cold distilled water and 1 mL 10 X PBS to lyse the contaminating erythrocytes (hypotonic lysis). The final solution was centrifuged at 1000 rpm for 10 min at room temperature. The pellet formed was then resuspended in 1 X PBS, and the cells were visualized using a 20 X objective lens and counted by trypan blue dye exclusion using a Neubauer hemocytometer. Cells were resuspended in Dulbecco’s Modified Eagle’s Medium (DMEM) with 3% FCS and kept on ice until further use.

### 2.5. In Vitro Model of Hypoxia and Normoxia

Transwell inserts with confluent HUVEC monolayers were kept for 20 h under hypoxic (2% O_2_, 5% CO_2_, and 73% N_2_) conditions (mimicking the ischemic phase in the IRI sequence), followed by 3 h under normoxic (21% O_2_, 5% CO_2_, and 74% N_2_) conditions (mimicking the early reperfusion phase in the IRI sequence), followed by the addition of FITC-dextran (10, 40, 70 kDa).

### 2.6. Neutrophil Transmigration

**Figure 1:**
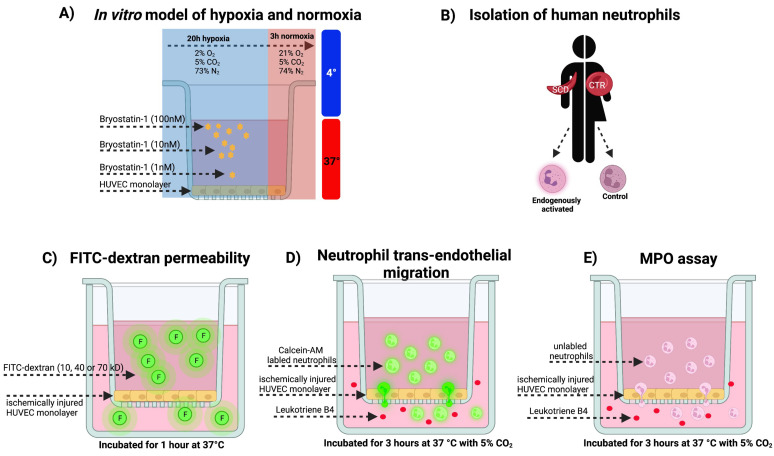
Experimental outline. (**A**) An in vitro model of hypoxia and normoxia was used. For this, transwell systems were equipped with human umbilical vein endothelial cell (HUVEC) monolayers and treated with varying concentrations of Bryostatin-1 (1, 10, 100 nM) or a vehicle control. Next, HUVECs were incubated for 20 h at 37° under hypoxic (2% O_2_, 5% CO_2_, and 73% N_2_) conditions (mimicking the ischemic phase in the ischemia reperfusion sequence), followed by 3 h under normoxic (21% O_2_, 5% CO_2_, and 74% N_2_) conditions (mimicking the early reperfusion phase in the ischemia reperfusion sequence). Control cells were kept under normoxic conditions. Some experiments were conducted under hypothermia (4°); (**B**) Human neutrophils were obtained from healthy volunteers (CTR) or sickle cell patients (SCD), a disease known for its endogenous neutrophil activation; (**C**) To study endothelial barrier function, control and ischemically injured HUVEC monolayers were kept in transwell systems, and FITC-dextran (either 10, 40, or 70 kD) was added to the upper chamber. After 1 h incubation, FITC-dextran concentrations in the lower chambers were measured using a plate reader; (**D**) To study neutrophil trans-endothelial migration, control and ischemically injured HUVEC monolayers were kept in transwell systems, calcein-AM labeled neutrophils were added to the upper chamber, and the chemoattractant leukotriene B_4_ (or vehicle control) was added to the lower chamber. After 3 h incubation, calcein fluorescence intensity was measured, and the migration index was calculated by dividing the number of neutrophils migrating toward LTB_4_ by the number of cells migrating to the vehicle. Subsets of experiments were conducted with neutrophils from SCD patients or after pre-treatment with tumor necrosis factor alpha; (**E**) To study neutrophil activation, control and ischemically injured HUVEC monolayers were kept in transwell systems, and unlabeled neutrophils were added to the upper chamber, and the chemoattractant leukotriene B_4_ (or vehicle control) was added to the lower chamber. After 3 h incubation, MPO activity was measured in the transmigrated neutrophils (this figure was created with BioRender.com, accessed on 25 February 2022).

After isolation, human neutrophils were tagged with Calcein-AM (Invitrogen, Waltham, MA, USA). Labeled neutrophils were diluted to a concentration of 10^6^ cells/mL and then treated with vehicle or compound and inserted at a concentration of 500,000 per insert on top of the HUVECS-containing transwell inserts. Each well was filled with either 500 μL of media (usually DMEM with 3% FCS) or leukotriene B4 (LTB_4_, 10^−6^ M) as a chemoattractant stimulus or PBS as a control. Plates were incubated for 3 h at 37 °C with 5% CO_2_ and then calcein fluorescence intensity was measured using a plate reader with 485/528 nm excitation/emission filter sets. Using the fluorescence data, a migration index was calculated by dividing the number of neutrophils migrating toward LTB_4_ by the number of cells migrating to the vehicle (Figure 1).

### 2.7. Myeloperoxidase (MPO) Assay

When unlabeled neutrophils were used, an MPO assay was conducted. Briefly, three solutions were prepared: solution 1 consisted of KH_2_PO_4_, EDTA and Triton-x-100 at pH 5.4, solution 2 was made up of 3,3′,5,5′-tetramethylbenzidine (TMB, 10 mM) (Sigma-Aldrich) prepared in acetone, and solution 3 contained 0.003% H_2_O_2_ in H_2_O. The final MPO assay solution was prepared by combining solutions 1, 2, and 3 in the ratio of 9:1:0.1, respectively. After three hours, the insert was removed, the 24-well plate was centrifuged at 1200 rpm for 10 min, and neutrophils were collected from the bottom of the well and added to the MPO solution at a 1:4 ratio, followed by incubation at room temperature until the color of the solution changed to blue, at which point H_2_SO_4_ (2 N) was added to terminate the reaction and absorbance was read at 450 nm (Figure 1).

### 2.8. FITC-Dextran Permeability Assay

After treatments, media were aspirated, and 500 μL DMEM with 3% FCS containing FITC-dextran (10, 40, or 70 kDa) was added into the upper chamber. Plates were incubated for 1 h at 37 °C. FITC-dextran concentrations in the lower chambers were measured using a plate reader with 485/528 nm excitation/emission filter sets (Figure 1).

### 2.9. Animals

A total of 14 animals (female German landrace pigs, 3–6 months of age) were used and kept in the central animal facility of the University of Münster with free access to water and standard chow during an acclimatization period of 7 days. All procedures were conducted in accordance with the German Animal Welfare Law and approved by the local animal care committee (Landesamt für Natur, Umwelt und Verbraucherschutz Nordrhein-Westfalen, permit reference number 84-02.04.2016.A21). Studies were performed blinded and randomized. Although 14 animals were randomized, one animal in the control group had to be terminated prior to the transplant because of suspected sepsis following graft retrieval. Body weights between both groups were comparable (Bryostatin-1: 36.4 ± 4.9 kg, placebo: 34.8 ± 6.0 kg, *p* = 0.63).

### 2.10. Experimental Design and Treatment Algorithm for In Vivo Studies

Animals were randomly assigned into the following groups: Bryostatin-1 (*n* = 7) and placebo (*n* = 7). To solely focus on early neutrophil infiltration in the sequence of renal ischemia reperfusion injury, a porcine kidney autotransplantation model [22] was used. This model excludes allogenic immune responses and omits acquired immune pathways through graft rejection, thus allowing selective investigation of innate immune responses. A graft-specific treatment algorithm was used, in which Bryostatin-1 (100 nM or an equivalent volume of PET in placebo animals) was added to 100 mL of standard perfusion solution during cold storage. Directly after graft retrieval, kidneys were flushed with 250 mL ice cold histidine-tryptophan-ketoglutarate (HTK, Dr F. Köhler Chemie, Bensheim, Germany). Subsequently, an additional 100 mL HTK (supplemented with Bryostatin-1 or placebo) was infused, and the renal vein and artery were sealed with vascular clamps to prevent outflow. Kidney grafts were stored at 4 °C for 20 h until implantation. The study was terminated 8 h after reperfusion, and the primary endpoint was the extent of neutrophil TEM. Punch biopsies of the kidney were collected at baseline (after cold storage), 30 min after reperfusion, and at the end of the experiment. Serum, plasma, and urine samples were collected at baseline (after cold storage), 30 min, and 4 and 8 h after reperfusion.

### 2.11. Anesthesia and Monitoring during Graft Procurement

After fasting for 12 h (with free access to water), animals were premedicated by intramuscular (i.m.) injection of azaperon (2 mg/kg b.w., Elanco, Bad Homburg vor der Höhe, Germany) and ketamine (15 mg/kg b.w., WDT eG, Garbsen, Germany). After the first venous access was established by cannulation of an ear vein, propofol (3 mg/kg b.w., Ratiopharm, Ulm, Germany) was used to initiate anesthesia, followed by endotracheal intubation. Maintenance of anesthesia was conducted using isoflurane (1.5 vol%, Baxter, Deerfield, IL, USA). Analgesia was ensured by continuous perfusion of fentanyl (0.005 mg/kg b.w., Rotexmedica, Trittau, Germany). Next, a central venous access catheter was placed in the right internal jugular vein and tunneled to the upper neck. Thirty minutes before incision, all animals received a single dose of i.v. antibiotic prophylaxis (Enrofloxacin, 5 mg/kg b.w., Bayer, Leverkusen, Germany). Animals were monitored by means of pulse, blood pressure, oxygen, and carbon dioxide levels, as well as body temperature (rectal probe) and intermittent blood gas analysis.

### 2.12. Graft Procurement

All surgical procedures were conducted under sterile conditions according to the general principles of surgical asepsis and antisepsis. Following a small median laparotomy (15 cm), the left kidney was recovered and transferred to the back table for the previously described treatment algorithm. Afterwards, the animals were weaned, extubated, and transferred to the housing facility to recover. Analgesia (Buprenorphine 0.05 mg/kg b.w., Indivior, Dublin, Ireland) was given every 8 h, and all animals received a single dose of a proton pump inhibitor (40 mg, Pantozol, Takeda, Konstanz, Germany). During the recovery phase, animals had free access to water and liquid food. Cold ischemia time (Bryostatin-1: 19 h 54 min ± 0.5, placebo: 19 h 40 min ± 0.6, *p* = 0.48) was similar between both groups.

### 2.13. Anesthesia and Monitoring during Autotransplantation

Induction of anesthesia was performed by using propofol (3 mg/kg b.w.) while maintenance of anesthesia was identical to the protocol during graft retrieval. For invasive blood pressure monitoring, the femoral artery was cannulated.

### 2.14. Orthotopic Kidney Autotransplantation

Following relaparotomy, the right kidney was removed, and the left kidney was implanted orthotopically by performing an end-to-end anastomosis of the renal vein (5-0 polypropylene, Covidien, Dublin, Ireland), as well as end-to-end anastomosis of the renal artery (6-0 polypropylene, Covidien). During reperfusion, mean arterial pressure was kept above 80 mm Hg by ensuring adequate volume management and partially by administration of norepinephrine (0.1–1.0 μg/kg b.w., Cheplapharm, Greifswald, Germany). Warm ischemia time (Bryostatin-1: 34.4 ± 4.4 min, placebo: 39.2 ± 13.6 min, *p* = 0.49) was comparable between both groups, and all thirteen transplanted grafts showed homogenous perfusion and direct diuresis immediately after reperfusion. In addition, directly after reperfusion, 100 mL 20% glucose solution was infused to stimulate initial osmotic diuresis. The graft ureter was splinted, tunneled through the right abdominal wall, and secured as ureterocutaneostomy. Lastly, the abdomen was closed, and the animals were kept under anesthesia until 8 h after reperfusion, after which all animals were sacrificed by a lethal dose of T61 (Intervet, Unterschleißheim, Germany).

### 2.15. MPO Assay for Tissue Samples

Briefly, tissue was weighed and suspended (10%, wt/vol) in 50 mM potassium phosphate buffer (KPi, Sigma-Aldrich, USA), containing 0.5% hexadecyl-trimethyl-ammonium-bromide (HETAB) (Sigma-Aldrich). Samples were first homogenized, then sonicated and subsequently centrifuged for 10 min at 12,000 rpm at 4 °C. For each sample, a mixture containing 2810 μL of KPi, 30 μL OD dihydrochloride (Sigma-Aldrich), and 30 μL hydrogen peroxide (20 mM, Sigma-Aldrich) was prepared. Instead of OD dihydrochloride, blanks contained 30 μL distilled water. The start of the reaction was induced by adding 100 μL of supernatant followed by an incubation time for 10 min at room temperature. The reaction was terminated by adding 30 μL 2% sodium azide (Fisher Scientific, Waltham, MA, USA), and the resulting change in absorbance 460 nm was read using a spectrophotometer (Biochrom Ltd., Berlin, Germany). MPO activity (U/g tissue) was expressed as the amount of enzyme necessary to produce a change in absorbance of 1.0 per minute per gram of wet weight of tissue.

### 2.16. Blood and Urine Analysis

Enzyme-linked immunosorbent assays (ELISA) were used to determine plasma levels of IL-8, TNF alpha (Invitrogen, USA), and Cystatin C (Cusabio, Wuhan, China), as well as urine levels of kidney injury molecule-1 (KIM-1) and neutrophil gelatinase-associated lipocalin (NGAL, MyBioSource, San Diego, CA, USA). All assays were performed according to the manufacturer’s instructions.

### 2.17. Histological Evaluation

Renal biopsies were fixed in 4% formaldehyde (Otto Fischer, Wiesbden, Germany), embedded in paraffin, sectioned, and stained with hematoxylin and eosin (H&E), as well as periodic acid-Schiff (PAS). Histological damage was assessed by light microscopy in sections of the cortex, medulla, and corticomedullary junction and evaluated by two board-certified pathologists blinded to the experimental conditions. Briefly, a previously published semiquantitative score was used [23] for evaluating glomerular damage (shrinking), inflammatory cell infiltrates, tubular damage, and edema. For each animal, four different sections were analyzed, and the extent of injury was graded on a scale from 0 to 5 (0 = no abnormality; 1 = mild, affecting <10% of the field; 2 = moderate, affecting 10–25% of the field; 3 = severe, affecting 25–50% of the field; 4 = very severe, affecting 50–75% of the field and 5 = extensive damage, involvement of >75% of the field).

### 2.18. Transmission Electron Microscopy

Renal biopsies were fixed in 2% (*v*/*v*) formaldehyde and 2.5% (*v*/*v*) glutaraldehyde in 100 mM cacodylate buffer, pH 7.4, at 4 °C. After washing in PBS, specimens were post-fixed in 0.5% (*v*/*v*) osmium tetroxide and 1% (*w*/*v*) potassium hexacyanofer-rate (III) in 0.1 M cacodylate buffer for 2 h at 4 °C, followed by intense washing with distilled water. After dehydration in an ascending ethanol series from 30 to 100% ethanol, specimens were incubated two times in propylene oxide each for 15 min. Next, small tissue pieces were embedded in Epon using flat embedding molds. Ultrathin sections were cut with an ultramicrotome, collected on copper grids, and negatively stained with 2% uranyl acetate for 15 min. Electron micrographs were taken with a Phillips EM-410 electron microscope using imaging plates (Ditabis, Pforzheim, Germany). All subsequent analyses of the ultrastructure of glomeruli were performed in a blinded fashion. Evaluation was focused on the structural integrity of the glomerular basement membrane, the podocytes, and the endothelium. Electron micrographs were evaluated and classified based on a 3-scale scoring system (1 = no or moderate damage; 2 = moderate damage; 3 = severe damage).

### 2.19. Quantitative Polymerase Chain Reaction (qPCR)

Total RNA was isolated from tissue samples using the RNeasy Mini Kit (Qiagen, Hilden, Germany) according to the manufacturer’s instructions. Afterwards, RNA was transcribed into cDNA by QuantiTect Reverse Transcription Kit (Qiagen), and cDNA was subsequently analyzed using QuantiTect SYBR Green PCR Kit (Qiagen). Next, qPCR was performed in a CFX384 real-time PCR Cycler (BIO-RAD, Germany). The gene expression of ICAM-1 (5′ GCTCAGTGTCCTGTATGGACC 3′), PECAM-1 (5′ CAGCAGCACCACTTCTGAAC 3′), TLR-4 (5′ CGTGCAGGTGGTTCCTAACA 3′), IL-8 (5′ TGCAGAACTTCGATGCCAGT 3′), and TNF alpha (5′ GTTGTAGCCAATGTCAAAGCCG 3′) was analyzed using Glyceraldehyde-3-phosphate dehydrogenase (GAPDH 5′ ACTTGGATGGGGTGGTCGTA 3′) as a housekeeping gene, which has proven to be reliable in pigs [24]. All primers were developed for the species *Sus scrofa* using the National Center for Biotechnology Information Primer Design Tool. It was ensured that the guanine-cytosine content was between 59 and 61% and that the primers had a length of 20–25 base pairs. In addition, the primers were not directly at the beginning or at the end of a respective gene. Next, the gene sequence was ordered commercially (Eurofins Genomics, Luxemburg). Results are presented as relative gene expression after calculation using the 2^−ΔΔC^T method.

### 2.20. Statistics

Statistical significance between two groups was analyzed using the unpaired Student’s *t*-test, whereas statistical significance between three or more groups was tested using ANOVA with Bonferroni post hoc testing. All data were analyzed with Graph Pad Prism (Version 8 or 9, Graph Pad Software, San Diego, CA, USA). Data are presented as mean values ± SEM, n values represent numbers of individually conducted experiments or animals, and *p* < 0.05 was considered statistically significant.

## 3. Results

### 3.1. Bryostatin-1 Reduces Hypoxia-Elicited Neutrophil TEM

**Figure 2:**
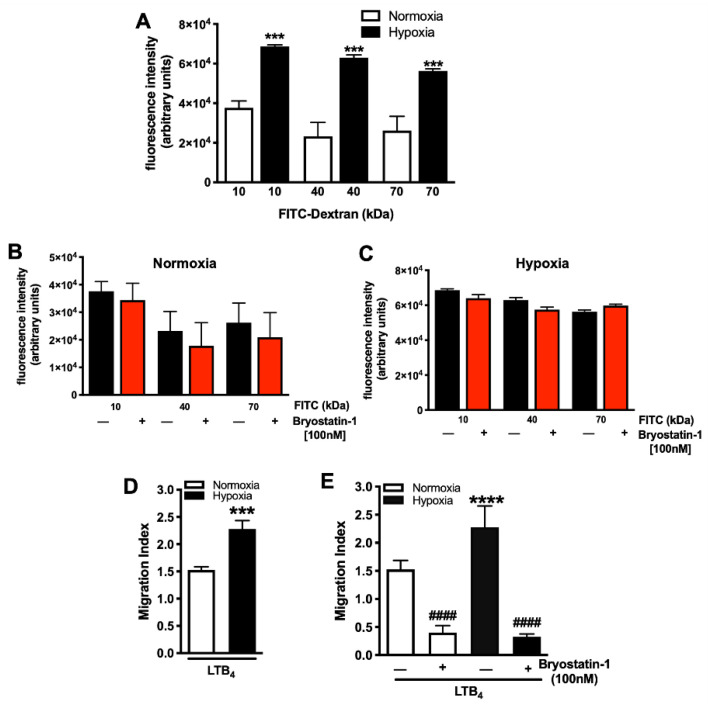
Bryostatin-1 prevents hypoxia-elicited endothelial permeability and blocks neutrophil transmigration. Confluent human umbilical vein endothelial cell (HUVEC) monolayers were subjected to either normoxia (21% O_2_, 5% CO_2_, and 74% N_2_) or hypoxia (2% O_2_, 5% CO_2_, and 73% N_2_) followed by normoxia, and (**A**) FITC-dextran (10 and 40 kDa) permeability was measured. Hypoxia induced a significant increase in HUVEC permeability for FITC, irrespective of the molecular size. (**B**) HUVEC monolayers were treated with 100 nM Bryostatin-1 or vehicle (1 X PBS), and permeability for FITC-dextran (10, 40, and 70 kDa) was tested under (**B**) normoxic or (**C**) hypoxic conditions. (**D**) Neutrophil transmigration across confluent HUVEC monolayers toward leukotriene B4 (LTB_4_, 10^−6^ M) was measured under normoxic and hypoxic conditions, showing a hypoxia-elicited increase in neutrophil transmigration. (**E**) Treatment with Bryostatin-1 (100 nM) blocked neutrophil transmigration under normoxic and hypoxic conditions. The migration index was calculated by dividing the number of calcein-AM labeled neutrophils that migrated to LTB_4_ by the number of cells that migrated to the vehicle. All results are representative of at least three independent experiments (n = 3–7). Graphs present mean ± SEM, and data were analyzed with ANOVA with Bonferroni post-tests (**A**–**C**,**E**) or Student’s *t*-test (**D**). Significance is indicated by the following symbols: (**A**,**D**) **** *p* < 0.0001 and *** *p* < 0.001 versus the respective normoxia group; (**E**) #### *p* < 0.0001 versus vehicle control.

When compared to control (normoxic) conditions, hypoxia elicited a significant increase in the permeability of HUVEC monolayers, irrespective of the molecular weight (Figure 2A). Next, we tested whether Bryostatin-1 (100 nM) could alter the permeability of the HUVEC monolayer. Figure 2B,C shows that the addition of Bryostatin-1 had no effect on FITC-dextran transport, either under normoxic (Figure 2B) or hypoxic (Figure 2C) conditions. Next, we wanted to address whether these findings with FITC-dextran held true for cellular transmigration over ischemically injured HUVEC monolayers and whether neutrophil TEM under normoxic and hypoxic conditions was tested. It was found that hypoxia was associated with a significant increased neutrophil TEM (Figure 2D). This effect of neutrophil TEM was significantly reduced in the presence of Bryostatin-1 under both normoxia and hypoxia (Figure 2E), suggesting a protective role for Bryostatin-1.

### 3.2. Dose-Dependent Effects of Bryostatin-1 in Altering Neutrophil Activation and Transmigration

**Figure 3:**
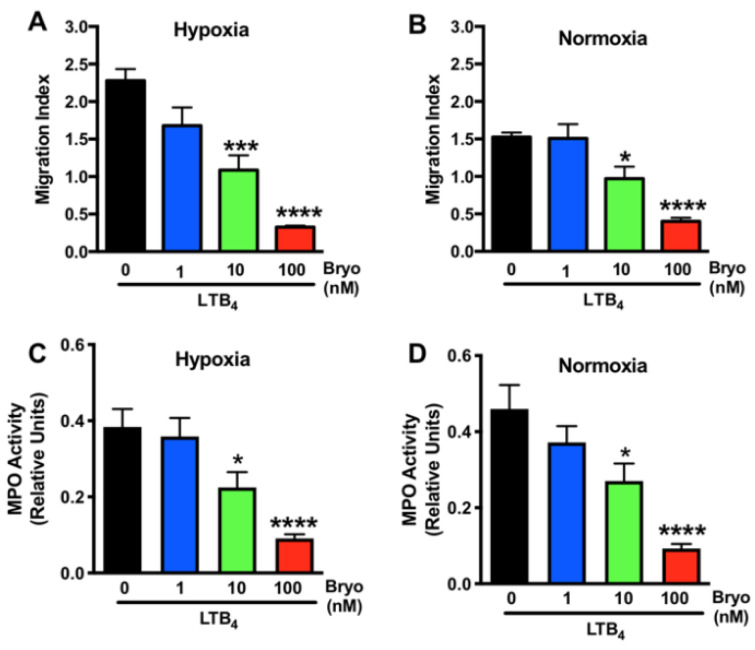
Dose-dependent effects of Bryostatin-1 in altering neutrophil activation and transmigration. Bryostatin-1 (Bryo) showed a dose-dependent (1, 10, 100 nM) effect on neutrophil activation as well as transmigration across confluent human umbilical vein endothelial cell (HUVEC) monolayers toward leukotriene B4 (LTB_4_, 10^−6^ M) under (**A**,**C**) hypoxic and (**B**,**D**) normoxic conditions. The migration index was calculated by dividing the number of calcein-AM labeled neutrophils that migrated to LTB_4_ by the number of cells that migrated to the vehicle. Neutrophil activation was determined by measuring myeloperoxidase (MPO) activity. All results are representative of at least three independent experiments (n = 5–7). Graphs present mean ± SEM, data were analyzed with ANOVA with Bonferroni post-tests, and significance is indicated by the following symbols: * *p* < 0.05 versus vehicle control, *** *p* < 0.001 versus vehicle control, **** *p* < 0.0001 versus vehicle control.

We next questioned whether the protective effects of Bryostatin-1 on neutrophil TEM were concentration dependent. Dosing studies were conducted, and a concentration-dependent effect of Bryostatin-1 in inhibiting human neutrophil TEM toward LTB4 across ischemically injured (hypoxic) HUVEC monolayers was revealed (Figure 3A). The same effect was found when HUVECs were kept under normoxic conditions (Figure 3B), and both sets of experiments established 100 nM Bryostatin-1 as the most effective dose. We further analyzed the influence of Bryostatin-1 on neutrophil activation by measuring MPO activity in transmigrated neutrophils. Again, Bryostatin-1 showed a dose-dependent effect in attenuating neutrophil activation, with 100 nM being the most effective dose under both hypoxic (Figure 3C) and normoxic (Figure 3D) conditions.

### 3.3. Bryostatin-1 Reduces Neutrophil TEM under Clinically Relevant Conditions

**Figure 4:**
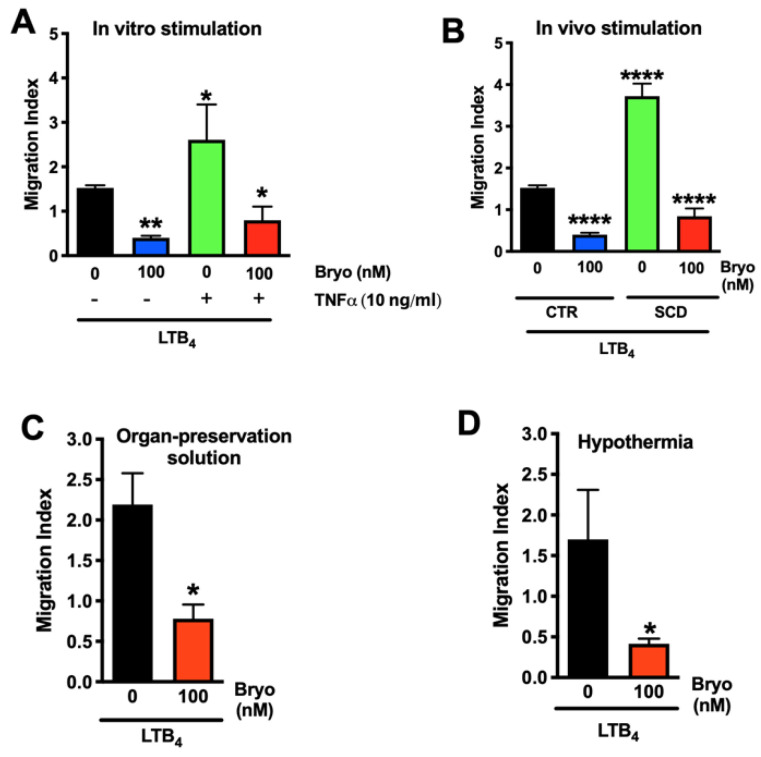
Bryostatin-1 reduces neutrophil transmigration under clinically relevant conditions. (**A**,**B**) Bryostatin-1 (Bryo, 100 nM) blocked hypoxia-elicited transmigration of stimulated neutrophils across confluent human umbilical vein endothelial cell (HUVEC) monolayers toward leukotriene B4 (LTB_4_, 10^−6^ M). (**A**) Neutrophils were stimulated in vitro by a 30-min treatment with tumor necrosis factor alpha (TNF-α). (**B**) Neutrophils were obtained from patients with sickle cell disease (SCD) and compared to neutrophils from control (CTR, healthy donors). (**C**,**D**) Bryostatin-1 (100 nM) remained effective in blocking hypoxia-elicited neutrophil transmigration across confluent HUVECs monolayers towards LTB_4_ even when (**C**) Bryostatin-1 was suspended in organ preservation solution (University of Wisconsin solution) or when (**D**) experiments were conducted under hypothermic conditions (4°). The migration index was calculated by dividing the number of calcein-AM labeled neutrophils that migrated to LTB_4_ by the number of cells that migrated to the vehicle. All results are representative of at least three independent experiments (n = 3–7). Graphs present mean ± SEM. Data were analyzed with ANOVA with Bonferroni post-tests (**A**,**B**) or Student’s *t*-test (**C**,**D**). Significance is indicated by the following symbols: * *p* < 0.05 versus vehicle control, ** *p* < 0.01 versus vehicle control, **** *p* < 0.0001 versus vehicle control.

To translate these in vitro findings to an in vivo setting, activated neutrophils were used to mimic activation of the host immune system after reperfusion. First, human neutrophils were pretreated with TNF-α, and then their ability to transmigrate across ischemically injured HUVEC monolayers was tested. Neutrophil activation with TNF-α (10 ng/mL) induced a significant increase in neutrophil TEM, which was blocked by Bryostatin-1 (100 nM) (Figure 4A). To further confirm these findings, neutrophils from SCD patients [21] were compared to neutrophils from healthy donors. Endogenous activation in SCD neutrophils elicited a significant increase in neutrophil TEM compared to controls (Figure 4B). Again, activation-elicited neutrophil TEM was blocked by Broystatin-1 (100 nM) (Figure 4B). To mimic the in vivo situation even more closely, experiments were conducted using the University of Wisconsin (UW) preservation solution, which is commonly used in human kidney transplantation [25] to deliver Bryostatin-1. Using UW solution had no influence on the effect of Bryostatin-1 on blocking neutrophil TEM (Figure 4C). To test the ability of Bryostatin-1 to be used as a therapeutic agent during the phase of static cold storage during kidney transplantation, Bryostatin-1 was used to pretreat HUVEC monolayers at 4 °C. We found that Bryostatin-1 was still able to block neutrophil TEM when tested under hypothermia (Figure 4D), indicating its potential usefulness during static cold storage in organ transplantation.

### 3.4. Bryostatin-1 Acts by Altering Neutrophil-Endothelial Cell Crosstalk Pathways

**Figure 5:**
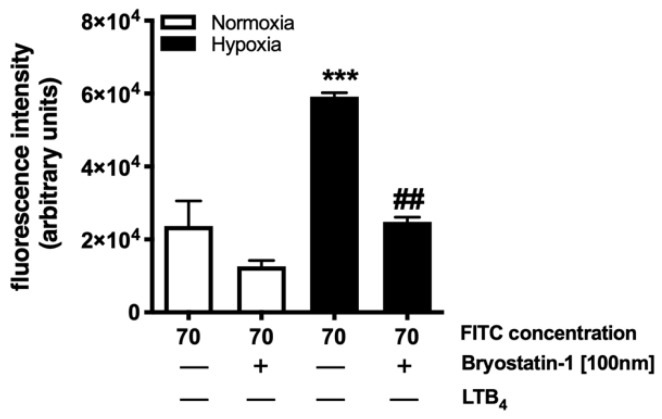
Bryostatin-1 acts by altering neutrophil–endothelial cell crosstalk pathways, rather than directly affecting the vascular endothelium. Confluent human umbilical vein endothelial cell (HUVEC) monolayers were treated with Bryostatin-1 (100 nM) or vehicle (1 X PBS), subjected to hypoxia or normoxia, followed by the addition of calcein-AM labeled neutrophils and leukotriene B_4_ (LTB_4_, 10^−6^ M). After three hours, neutrophils were removed, FITC-dextran (70 kDa) was added, and permeability was measured. Bryostatin-1 significantly reduced permeability for FITC-dextran. The migration index was calculated by dividing the number of calcein-AM labeled neutrophils that migrated to LTB_4_ by the number of cells that migrated to the vehicle. All results are representative of at least three independent experiments (n = 7). Graphs present mean ± SEM, data were analyzed with ANOVA with Bonferroni post-tests, and significance is indicated by the following symbols: *** *p* < 0.0001 versus the respective normoxia group; ## *p* < 0.001 versus vehicle control.

Having discovered that Bryostatin-1 had no effect on HUVEC monolayer permeability for FITC-dextran (elicited by hypoxia) but was highly effective in blocking neutrophil transmigration across ischemically injured HUVEC monolayers, we next ascertained whether the influence of Bryostatin-1 neutrophil–endothelial cell crosstalk would affect monolayer permeability. Confluent HUVEC monolayers were treated with Bryostatin-1 or vehicle and subjected to hypoxia or normoxia. Neutrophils were then added for three hours, after which they were aspirated, and FITC-dextran was added. Here, we found that Bryostatin-1 significantly reduced permeability for FITC-dextran following hypoxia (Figure 5). Collectively, these novel findings suggest that the protective effects of Bryostatin-1 are likely based on neutrophil–endothelial interactions rather than direct effects on the vascular endothelium itself.

### 3.5. Bryostatin-1 Attenuates Neutrophil Transmigration in Renal Autografts

**Figure 6:**
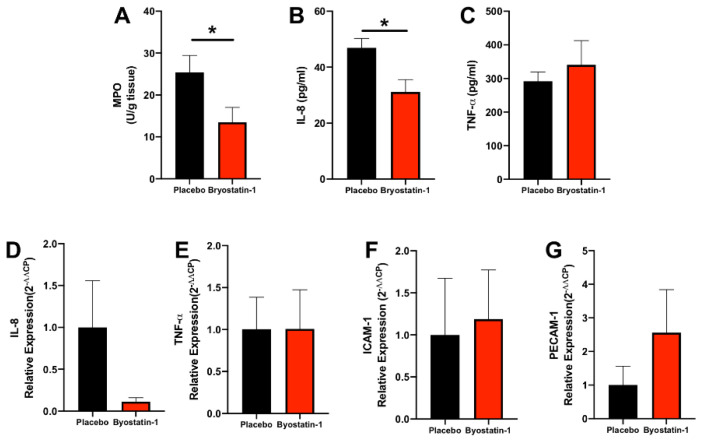
Bryostatin-1 attenuates neutrophil transmigration in renal autografts. All analyses were conducted eight hours after reperfusion in a porcine model of renal autotransplantation with graft-specific treatment with placebo or Bryostatin-1 during 20-h static cold storage of the renal autografts. (**A**) Myeloperoxidase (MPO) activity (in units per gram renal tissue) in renal autografts was measured as an index for tissue neutrophil content and was significantly reduced in Bryostatin-1 treated renal autografts. (**B**) Systemic protein concentration (pg/mg) of interleukin-8 (IL-8) was significantly reduced in Bryostatin-1 treated animals, while (**C**) protein concentration (pg/mg) of tumor necrosis factor alpha (TNF-α) was comparable in placebo and Bryostatin-1 groups. To correlate systemic protein levels with local tissue changes, gene expression of (**D**) IL-8 and (**E**) TNF-α was analyzed in renal biopsies. Gene expression of cellular adhesion molecules (platelet endothelial cell adhesion molecule-1 (PECAM-1) (**F**) and intercellular adhesion molecule 1 (ICAM-1) (**G**)) was tested and found to be comparable between groups. All data are presented as mean values ± SEM from 6 (placebo) or 7 (Bryostatin-1) individually analyzed animals per group. Data were analyzed with Student’s *t*-test, and significance is indicated by the following symbol: * *p* < 0.05 versus placebo.

To translate our in vitro findings into a clinical setting, we performed a porcine kidney autotransplantation model. Primary outcome endpoints in the in vivo porcine study were the extent of neutrophil TEM and the blockade of neutrophil influx into ischemically damaged grafts by Broystatin-1. To assess these effects, an MPO-assay was used to quantify the neutrophil burden in renal biopsies after 8 h of reperfusion. It was found that graft-specific Bryostatin-1 treatment significantly reduced MPO activity (correlating with neutrophil activation as a surrogate marker for neutrophil TEM) (Figure 6A). Having demonstrated the therapeutic effect of Bryostatin-1 on neutrophil TEM in the sequence of renal IRI, we next investigated whether it would also ameliorate systemic inflammation. Levels of TNF-α and IL-8 were analyzed because both have been characterized not only as proinflammatory mediators in renal IRI but also have been associated with neutrophil activation and recruitment. Bryostatin-1 elicited a significant reduction in IL-8 (Figure 6B), while systemic concentrations of TNF-α remained unchanged (Figure 6C). To correlate systemic protein levels with local tissue changes in gene expression, mRNA production of IL-8 and TNF-α were analyzed in renal biopsies after 8 h of reperfusion. The results followed the systemic trend, with a reduction of IL-8 in Bryostatin-1 treated animals (Figure 6D); however, this did not reach statistical significance. The gene expression of TNF-α was comparable between the groups (Figure 6E). Since previous reports suggested that Bryostatin-1 blocks neutrophil TEM by inducing endothelial PKCδ activation rather than changes in adhesion molecules, the expression of two established cellular adhesion molecules (platelet endothelial cell adhesion molecule-1 (PECAM-1) and intercellular adhesion molecule 1 (ICAM-1)) was assessed. Similar to our previous studies [18], gene expression of both molecules was comparable between the two groups (Figure 6F,G).

### 3.6. Bryostatin-1 Protects Renal Autografts from Ischemia-Reperfusion Injury

**Figure 7:**
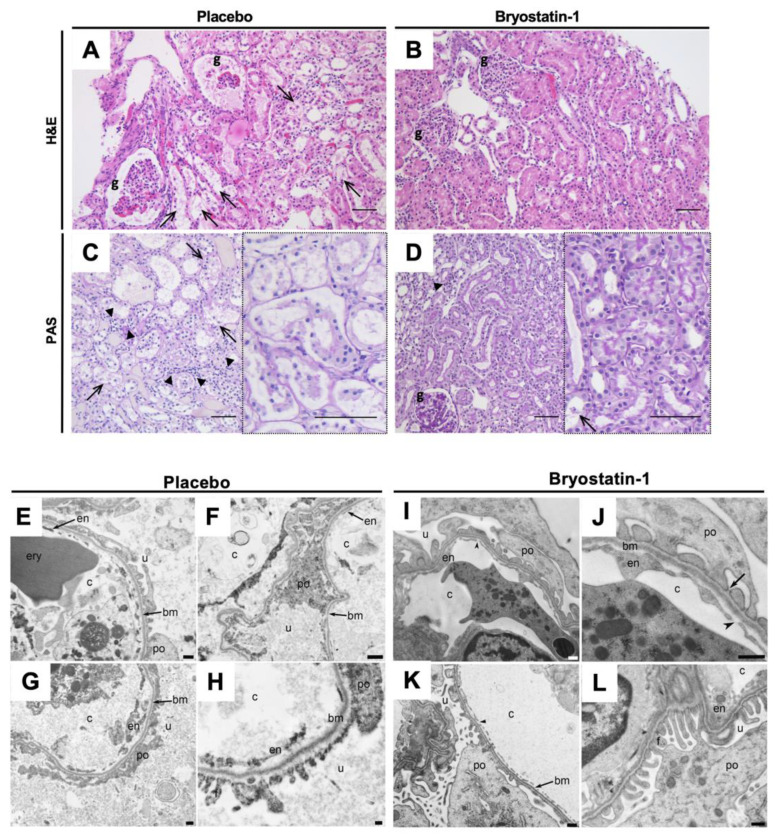
Protective effect of Bryostatin-1 following renal autotransplantation. All analyses were conducted eight hours after reperfusion in a porcine model of renal autotransplantation with graft-specific treatment with placebo or Bryostatin-1 during 20-h static cold storage of renal autografts. (**A**–**D**) Representative histopathologic images of hematoxylin and eosin (H&E), as well as periodic acid-Schiff (PAS), stained renal biopsies after eight hours of reperfusion: Samples from placebo-treated renal autografts (**A**,**C**) display striking glomerular shrinkage, distinct inflammatory cell infiltrates (arrowheads), and extensive tubular damage (arrows and (**C**) right panel), all of which were markedly reduced in the Bryostatin-1 group (**B**,**D**). g: glomeruli, bars: 50 µm. Ultrastructural injury score (consisting of structural integrity of glomeruli, basement membrane, podocytes, and endothelium) was significantly improved in Broystatin-1 treated renal autografts eight hours of reperfusion. Representative electron micrographs of (**E**–**H**) placebo and (**I**–**L**) Bryostatin-1 treated renal autografts: (**E**) Transmission electron microscopy in placebo treated renal autografts reveals ultrastructural changes of the endothelium (em) with massive disruptions of the endothelial integrity. Note that the endothelial fenestrations are not distinguishable due to tissue disintegration. In addition, (**F**,**G**) podocytes (po) and foot processes (f) showed irregular contours with destruction and flattening of the foot processes. As seen in (**H**), the basement membrane (bm) shows an irregular thickness. In comparison to control samples (placebo), (**I**) capillaries from Bryostatin-1 treated samples revealed an intact endothelium with (**J**,**K**) regular contours and multiple clearly distinguishable fenestrations. Podocytes showed a normal morphology with an intact cytoplasm and clearly visible organelles. (**L**) Foot processes with slit diaphragms and basement membranes reveal all characteristics of a normal glomerular filtration unit. Scale bar for E, G, I, J, K 500 nm, for F, H, 200 nm and for L 1 µm; c = capillary lumen, ery = erythrocyte, u = urinary space, arrow = slit diaphragms; arrowhead = fenestration.

**Figure 8:**
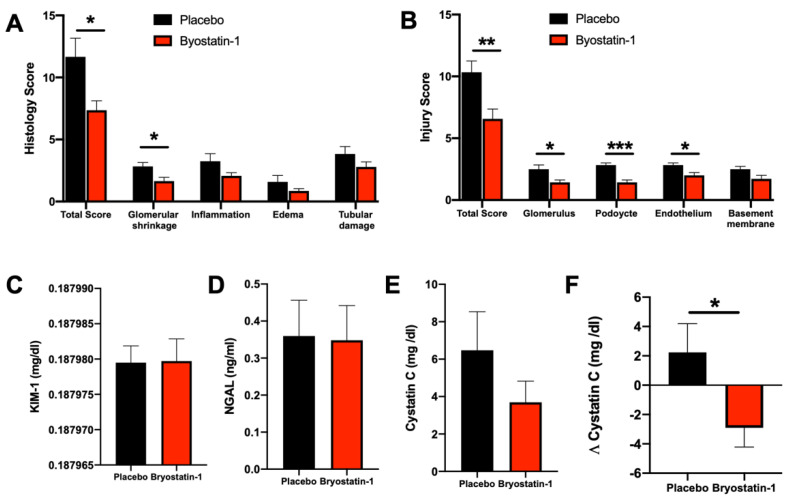
Bryostatin-1 protects renal autografts from ischemia-reperfusion injury and improves kidney function following renal autotransplantation. All analyses were conducted eight hours after reperfusion in a porcine model of renal autotransplantation with graft-specific treatment with placebo or Bryostatin-1 during 20-h static cold storage of renal autografts. (**A**) Histological injury score (consisting of glomerular damage (shrinking), inflammatory cell infiltrates, edema, and tubular damage) was significantly reduced in Broystatin-1-treated renal autografts. (**B**) Ultrastructural injury score (consisting of structural integrity of glomeruli, basement membrane, podocytes, and endothelium) was significantly improved in Broystatin-1 treated renal autografts eight hours of reperfusion. Urine levels of (**C**) kidney injury molecule-1 (KIM-1) and (**D**) neutrophil gelatinase-associated lipocalin (NGAL) were measured eight hours after reperfusion in a porcine model of renal autotransplantation with graft-specific treatment with placebo or Bryostatin-1 during 20-h static cold storage of renal autografts and found to be comparable between the two groups. (**E**) Plasma levels of Cystatin C were measured after 8 h of reperfusion, with a non-significant reduction in animals receiving Bryostatin-1 treated renal autografts. (**F**) When the individual delta (difference between pre-transplant and eight-hour reperfusion sample) was calculated, Bryostatin-1 elicited a significant reduction in ischemia-associated rise in plasma Cystatin C. All data are presented as mean values ± SEM from 6 (placebo) or 7 (Bryostatin-1) individually analyzed animals per group. Data were analyzed with Student’s *t*-test, and significance is indicated by the following symbols: * *p* < 0.05 versus placebo, ** *p* < 0.01 versus placebo, *** *p* < 0.001 versus placebo.

Next, we examined whether reduced neutrophil TEM in Bryostatin-1 treated grafts would affect cellular injury (the hallmark event of renal IRI), as evidenced by histological damage. Histological examination of cortical biopsies of Bryostatin-1 treated grafts showed a significant reduction in overall injury (Figure 7 and Figure 8A). This was evident by an attenuation in glomerular shrinkage, edema, inflammation, and tubular damage (Figure 7A–D). To further analyze morphological differences between placebo and Bryostatin-1 treated kidneys, transmission electron microscopy was used to evaluate IRI-elicited ultrastructural alterations (Figure 7E–L and Figure 8B). While the placebo group revealed evidence of severe ultrastructural damage, samples from Bryostatin-1 treated animals showed the characteristic morphology of normal glomeruli. The preserved structure was evident in the podocyte morphology, with finger-like extensions covering the glomerular capillary walls. Organelles, such as the endoplasmic reticulum and mitochondria, were visible and normal appearing in the cytoplasm. Moreover, the filtration cleft was clearly visible and regularly bridged by fine fibrillar structures, the slit diaphragms. The basement membrane showed a homogeneous ultrastructure, and the endothelial lining with fenestrations displayed all characteristics of a normal glomerulus. Conversely, podocytes in the placebo group lost this characteristic morphology, visible by irregular contours in combination with changes in the interdigitating foot processes. The cytoplasm appeared diffuse, and organelles were no longer clearly visible. Moreover, the podocyte foot processes were flattened and partly detached from the basement membrane. In addition, the basement membrane was more diffuse and heterogeneous in thickness. The endothelial lining was disrupted with irregular cell contours, and fenestrations were no longer distinguishable.

### 3.7. Bryostatin-1 Improves Kidney Function following Renal Autotransplantation

Having demonstrated a protective effect of graft-specific Bryostatin-1 treatment in terms of reduced neutrophil TEM, changes in pro-inflammatory markers of neutrophil activation and recruitment, and reduced histological injury, we next tested whether this would translate to kidney-specific markers of injury. Analyzing urine samples, we found that both KIM-1 (biomarker of tubular damage) and NGAL (biomarker of acute kidney injury) were comparable between the two groups (Figure 8C,D). Next, serum levels of Cystatin C (a sensitive marker for acute changes in renal function) were analyzed. After 8 h of reperfusion, a trend toward a reduction in the Bryostatin-1 group was noted; however, this did not reach statistical significance (Figure 8E). When the individual delta (difference between pre-transplant and 8-h reperfusion sample) was calculated, a significantly smaller ischemia-associated rise of the cystatin C serum levels in the Bryostatin-1-treated group was found (Figure 8F).

## 4. Discussion

Mounting evidence suggests a superior role of IRI in determining short- and long-term graft fate, which positions IRI as the ideal target for novel therapies in organ transplantation. The present study demonstrates for the first time that activation of PKCδ using Bryostatin-1 confers striking protection against renal IRI by blocking neutrophil TEM. We provide direct in vitro, as well as in vivo, evidence that the macrolide Bryostatin-1 is able to prevent the IRI-associated hallmark event of neutrophil TEM in kidney grafts and ameliorate subsequent histological damage, as well as renal function.

IRI is known to elicit a robust, rapid, and prolonged transmigration of circulating neutrophils into the interstitial compartment of the kidney [11]. Infiltrating neutrophils contribute to graft damage through the release of ROS [26], proteases (e.g., neutrophil elastase [27]), metalloproteinases (MMP, especially MMP-9), and the formation of neutrophil extracellular traps [28]. The dominating role of neutrophils in renal IRI is further underlined by the finding that renal MPO activity correlates with plasma creatinine, demonstrating a direct link between neutrophil infiltration and renal function [29]. Our results of reduced renal MPO and attenuated histological damage by Bryostatin-1 administration indicate a promising role of Bryostatin-1 in neutrophil-targeted therapies against IRI. In addition, mounting evidence now points to an additional role of neutrophils integrating the innate and acquired immune response in transplant immunology. Since neutrophils drive the recruitment of alloreactive CD8+ T cells [30], decrease allograft acceptance [31], and are involved in antibody-mediated and chronic rejection [32], Bryostatin-1 has significant potential to further influence long-term graft survival by ameliorating IRI and enhancing alloimmunity.

In addition to the clearly demonstrated mechanism of action to prevent neutrophil TEM across ischemically damaged endothelial monolayers in vitro and into engrafted kidneys in vivo, several other lines of evidence suggest additional effects of Bryostatin-1. Bryostatin-1 has been shown to reduce oxidative stress and ameliorate free radical-associated cell death [33]. In addition, Bryostatin-1 can also downregulate MMP-9 expression [34]. Most importantly, various immunomodulatory pathways have been thoroughly established for Broystatin-1, including the promotion of an anti-inflammatory phenotype in dendritic cells and macrophages [35,36].

Our study provides further evidence that the mechanism of action for Bryostatin-1 in blocking neutrophil TEM appears to be independent of changes in adhesion molecule expression. Our findings that the expression of ICAM-1 and PECAM-1 levels remained unchanged in Bryostatin-1 treated groups are consistent with earlier data [18]. We previously showed that Bryostatin-1 had no influence on ICAM-1, PECAM-1, or integrin-associated proteins in vitro [18], which was additionally confirmed in vivo. We have now also added new evidence to the field by building on our previous foundations regarding Bryostatin-1′s lack of influence on endothelial barrier integrity. For the first time, we showed that Bryostatin-1 alters HUVEC permeability following neutrophil-endothelial interactions, which was not the case under ischemic or normoxic conditions. This may also account, at least in part, for the recent findings of Awad et al., who described a correlation of neutrophil infiltration after renal IRI and increased vascular permeability [11]. It is therefore reasonable to assume that the mechanism by which Bryostatin-1 blocks neutrophil TEM is not solely mediated by acting on resting ECs but rather involves suppressing neutrophil-dependent junctional disintegration. Based on our current and previous data, it appears most likely that these cascades could include PKCδ-dependent phosphorylation of endothelial focal adhesion kinase tyrosine [18].

It is notable that our data provide no direct evidence regarding neutrophil adhesion, which in part could be responsible for the remaining graft injury in the Bryostatin-1 treated group. Neutrophil adhesion to the endothelium is known to elicit severe damage in the sequence of renal IRI through capillary plugging and vascular congestion [37]. Bryostatin-1 failed to reduce LTB4-induced neutrophil adhesion to human microvascular endothelial cells and HUVECs [18]. Thus, neutrophil-endothelial cell adhesion may play an important role in nephron destruction [38] by hindering oxygen and nutrient delivery, all of which are not influenced by Bryostatin-1.

PKCδ is a key mediator for a variety of cellular pathways associated with numerous biological functions. In the context of IRI, involvement of PKCδ in regulating redox signaling and oxidative stress, intracellular calcium overload, cell death (necrosis and apoptosis), and mitochondrial function are of special interest [39]. Although extensively studied, there is still an ongoing controversy regarding the role of PKCδ in IRI-elicited injury. Inagaki et al. found a selective inhibition of PKCδ to be protective in a porcine model of cardiac IRI [40], as well as in mice, isolated rat hearts, and ex vivo tested cardiac myocytes [41,42]. In addition, Chou et al. found PKCδ-null mice to be protected from cerebral IRI [43].

In contradistinction to these studies, activation of PKCδ has been shown to be involved in protective signal cascades during ischemic preconditioning and liver IRI, mainly through suppression of hepatocellular necrosis [44]. Similar results were obtained in models of cerebral IRI, where Bryostatin-1 induced antiapoptotic and synaptogenetic pathways through PKCδ activation [45,46]. Our results differ from those of Zhu et al., who described activation of PKCδ as associated with IRI-elicited mitochondrial damage, tubular cell death, and suppression of tubular repair mechanisms in the kidney [47]. However, it should be noted that mechanisms of action for PKCδ are highly tissue specific and even depend on its cellular localization. Our in vivo, as well as in vitro, data have focused on endothelial PKCδ and the form of delivery (via the vasculature during cold storage) is most likely to be associated with an activation of endothelial PKCδ.

## 5. Conclusions

In conclusion, our study provides substantial evidence that Bryostatin-1 is a promising pharmacological candidate for graft-specific treatment in kidney transplantation. Our findings also have the potential to be translated to other organs in the field of transplant medicine.

## 6. Patents

U.S. Patents No. 9,994,585, 12 June 2018 and No. 10,723,744, 28 July 2020, ‘Transplantation Therapies’, J. Steven Alexander (Shreveport, LA, USA, LSU, Shreveport, LA, USA, April C. Carpenter, Cincinnati, OH, USA and Trevor P. Castor, Arlington, MA, USA, Aphios Corporation, Woburn, MA, USA [48,49,50].

## Data Availability

Not applicable.
